# Progress in Cancer

**Published:** 1902-04-26

**Authors:** 


### Progress in Cancer.

The hopefulness of Beatson's treatment of carcinoma mammæ by oöphorectomy is not standing the test of time according to recent publications. One of Beatson's cases, which was supposed at the time to be bearing out his views on the advantage of this procedure, is reported now by McNicol to have died of the original disease 11 months after the operation.[1]

At first the cancerous deposits and nodules disappeared with the exception of glands above the clavicle, but eventually they all reappeared as before, together with evidence of secondary growths in various parts of the body. These reappearances became evident at the end of six months, and the remaining five months the patient is described as enduring a "long drawn out, painful, and living death." McNicol thinks that the treatment may be of value in early cases, but not in hopelessly inoperable cases.

The history of all published cases of oöphorectomy for recurrent carcinoma mammæ has been reviewed by Butlin,[2] who finds that there is no case which has been permanently cured by this operation. He regards the operation for the removal of the breast as offering two chances (1) the possibility of cure; (2) the exchange of the very painful and distressing condition in the breast for the less painful and distressing condition of recurrent cancer elsewhere. He gives the report of a case where he removed a breast for the relief of pain—this was so satisfactory that the patient returned for the removal of the other, which had become infected. In two other cases, one of which was operated upon, the other not, the report of a relative was that while the latter suffered grievously, the former died a much less miserable death. In the light of such experience as this, therefore, he does not admit the advisability of performing oöphorectomy in place of removal of the breast, as has been suggested by Beatson.

In the consideration of possible coincidence of improvement following oöphorectomy Pearse[3] records a case of inoperable cancer of the breast, with paroxysmal cough and cachexia, whose term of life was considered to be at the most three months. Oöphorectomy was considered, but set aside, but yet the patient lived for a year after. Not only is this operation stigmatised as unnecessary mutilation, but also several writers speak adversely to the extensive operations of Halsted and others, in which the pectoral muscles are removed with the glands, not only of the axilla, but the supra-clavicular set too. Sir William Banks[4] states that not only is this already extensive operation rendered much more formidable, but his experience tends to show—and in this he is borne out by Plimmer—that recurrence occurs, not in the great pectoral muscle and in the tissues between it and the small one, but in the skin and adjacent fat. He knows of no one who has lived long after the neck glands have become involved. The operation is enough to terrify the patients from coming with operable cancers for removal.

Dr. Herbert Snow[5] points out similar reasons for not encouraging these extensive operations. He points out that in scirrhous carcinoma the secondary infection of glands occurs within 12 weeks after the inception at the outside, and often within six. A scirrhus commencing at the sternal end of the mamma will early infect the lymphatic material underlying the sternum, including the remains of the thymus and the mediastinal glands. And so the most radical operation is valueless.

The lymph glands show three stages of infection: (1) They remain latent for several weeks; (2) then they show a tenderness on pressure, without enlargement, in about 14 days; (3) then they become palpably increased in size. The lymph circulation now becomes impeded and regurgitation occurs into the surrounding parts, including the marrow of the bones, humerus or sternum, which leads to neuralgic pain and sometimes to enlargement, and rarely fracture of these bones. The gnawing lumbar pain is due to the vertebræ becoming involved in the later general infection, usually at the end of the third or fourth year. This infection of the medulla of bones is very insidious and may take not a few years to become apparent, but all patients of mammary carcinoma receive it as an immediate sequela to axillary gland enlargement. This only shows how futile Halsted's operation and its developments are in relation to cancer, when this has once become at all spread. Such treatment can only do harm by lowering the general vitality and does not save life. Chronic widespread deposits are often attended by longer life than the solitary growth rapidly growing in a young and vigorous patient. And once they get under the influence of opium in moderate doses "the tenacity to life is amazing."

Opium administration from the first without operation will generally prevent ulceration and compel the disease into an atrophic stage. Previous ulceration forcibly militates against opium. It is wrong to remove supra-clavicular glands under almost any conditions, as deposit in these glands signifies deposits in the inaccessible mediastinal glands. In operating the efforts should be directed to the removal of the subcutaneous tissue round the mammary parenchyma and complete evacuation of the axilla. Recurrence of an average case of carcinoma within less than two years means inefficient work. The three years' period of immunity of Volckmann is absurd, as the marrow deposits very often make no sign till after that, or even one or two years later. After operation for mammary carcinoma all women require supervision during the remainder of their lives, and the majority should henceforth take small doses of opium habitually.

Not much is brought forward in support of the parasitic theory. Dr. Buchanan[6] shows a specimen of Tradescantia virginica which reveals various stages of cell divisions, and he points out the resemblance of many nuclei to cancer bodies. He urges the importance of the centrosome in cell division and the possibility that cancer bodies may be an expression of an exaggerated or abnormal condition of the centrosome. This may be hereditary. His belief is in the parasitic origin of cancers, and that it is more closely associated with a raw vegetable than a meat diet. His attention has been drawn to the presence about the houses of certain cancer patients addicted to salads in their diet, of remarkable numbers of snails. That the exclusive vegetable diet is not incompatible with cancer would appear from the correspondence between Roger Williams[7] and certain authorities in India. In some places it is said to be as frequent as elsewhere, though Keetley upholds his statement that Hindoos never suffer from cancer of the stomach.

In searching for an extrinsic cause of malignant tumours, Dr. C. Powell White[8] considers that a similar causation should be found for benign tumours at the same time. He has studied tumours from the histological and pathological standpoints with the object of finding out whether their formation can be explained by a parasitic theory or not. Amongst his many objections to this are :—(1) That unlike any other known inflammatory condition caused by irritants, carcinoma is apparently welcomed by the body: there being no true defensive reaction. (2) There is no statement as to whether the parasite of carcinoma differs from that of sarcoma, or whether they are due to the same parasite. If they differ it would be reasonable in carcinoma to expect to find the metastatic growths differing according to the kind of epithelium in which they appear. If they are the same, how is it that a primary carcinoma does not cause a secondary sarcomatous deposit? If each kind of tumour has its own parasite, then there would have to be a series of parasites, each of which is only capable of acting upon one kind of cells, and therefore one kind for each gland in the body and a different series for each species of animal!

Those cells infected by known parasites undergo degeneration and do not proliferate; according to this the cells which are carried from carcinomatous tumours cannot contain the parasite, and so we are reduced to believing that for the formation of metastatic growths a parasite and a cell are carried simultaneously to the same spot. From these and other arguments he concludes that the cause cannot be an extrinsic one. Of intrinsic theories, he finds that of Ribbert the most satisfactory, which suggests that tissues normally are prevented from overstepping their natural bounds by the action of the tissue tension of the part. When, by inflammation, etc., a cell or group of cells gets isolated from its surroundings, then it grows unrestrained by its natural boundaries. He finds fault with Adami's theory in that he offers no explanation of the phenomenon of penetration in cancer, which is its essential lesion. He considers also that there is equal justification for the statement that the cell ceases to functionate because it is proliferating, while Adami states that it either must functionate or proliferate, the latter occurring when it no longer can utilise in functionating assimilated material.

Imperfect drainage and soaking of the subsoil is offered as the cause of the high percentage of the infection of the alimentary system by cancer, according to the statistics of the Leamington district, by Harold Mason.[9] Women and old people are more likely to be infected because they are more tied to the house, and so longer exposed to the infection.

Warthin[10] has recorded a case of multiple sarcoma and adeno-carcinoma of the pyloric end of the stomach occurring in the same patient. The patient was treated by Coley's fluid, and rapid death ensued. He thinks that this agent may be dangerous in hastening cachexia and in loosening cells into the general blood system.

A large number of the subjects of cancer are found by Leser[11] to present numerous small angiomatous cutaneous tumours of a bluish colour. They are raised from the skin, and appreciated best by passing the hand lightly over the surface. Their location bears no relation to the course of the blood-vessels or nerves. They are present in other normal persons, but never in so large a number, and so he considers that they may prove a diagnostic sign in persons under 50 years of age. As many as 58 to 76 have been seen in single instances.

A very complete record of a case of that rare affection termed chloroma or green cancer has been given by Dr. Melville Dunlop.[12] Of this only 24 cases have been recorded, and this is claimed to be the first in which a diagnosis was made before death. A boy aged five years, born healthy, began to show weakness and swelling of the face on September 30th, without obvious cause. The history revealed that the patient had been subject to ecchymoses, attributed to bruises. Five months previously he was noticed to be very pale, and showed great tendency to suppurate on slight abrasions. Four months previously he had got wet and had never got over it. Pallor increased with loss of appetite and emaciation, exophthalmos ensued and deafness, an abscess of the scalp and a large number of petechial hæmorrhages over the body, and two larger ones over the vertebral column and right knee. The face was pale, pasty, and eyelids puffy, especially the left. There was deficiency in the red corpuscles and hæmoglobin, while the leucocytes were in excess, the lymphocytes preponderating to 73 per cent. A hard, indurated, purple swelling appeared on the hard palate, and a smooth rounded tumour in each temporal region. Chloroma was diagnosed at this point. Later, the eyelids were involved, hæmorrhages in the retinæ with optic neuritis, and then a pale greenish yellow tumour of a jelly-like consistence appeared at the inner canthus of the right eye, advancing over the eyeball. The left eye became similarly affected with ensuing frontal neuralgia and photophobia. The corneæ were completely covered and destroyed. The anæmia was now profound: red corpuscles=815,000, hæmoglobin 12 per cent., while the leucocytes numbered 107,000. Death ensued on November 16th, 47 days after admission. The diagnosis was confirmed post-mortem, a large number of deposits of bright green tissue were found in the bones, kidneys, viscera, muscles, etc., leaving out, however, the brain and spinal cord. The green colour faded on being exposed to air or being placed in water. This had been explained as being due to minute molecular granulations possessing great refractive power placed between the cells. But Dr. Dunlop thinks that the colour is more probably due to some chemical product allied to lipochrome, which loses its colour by oxidation. The original source of the tumours is admitted to be the periosteum of the head and face, and secondary only in other organs. The consistency of the tumours varied from being soft and gelatinous to being hard and gelatinous: microscopically consisting of large round or oval-shaped cells slightly larger than white corpuscles embedded in intercellular amorphous material of clear transparent nature or in a delicate network; the highly refractile granules lying in between the cells. The prognosis is invariably fatal, the average duration being five to six months; some cases have lasted above two years.

[1] Brit. Med. Jour., Nov. 9, 1901. [2] Brit. Med. Jour., Jan. 4, 1902. [3] Ibid. [4] Ibid. [5] West London Med. Jour., Oct. [6] Brit. Med. Jour., Feb. 1. [7] Lancet, Jan. 18. [8] Lancet, Feb. 15. [9] Brit. Med. Jour., Jan. 18. [10] Med. Rec., Nov. 2. [11] Med. Rec., Jan. 11. [12] Lancet, Feb. 15.

